# Preoperative chest radiographs in hip fracture patients: is there any additional value?

**DOI:** 10.1007/s00590-017-1971-3

**Published:** 2017-05-11

**Authors:** Sverre A. I. Loggers, Georgios F. Giannakopoulos, Edwin Vandewalle, Micha Erwteman, Ferco Berger, Wietse P. Zuidema

**Affiliations:** 1 Department of Trauma Surgery, VU University Medical Centre, 7F029, De Boelelaan 1117, 1007MB Amsterdam, The Netherlands; 2 Department of Emergency Medicine, VU University Medical Centre, Amsterdam, The Netherlands; 3 Department of Anesthesiology, VU University Medical Centre, Amsterdam, The Netherlands; 4 Department of Radiology, VU University Medical Centre, Amsterdam, The Netherlands; 5 Department of Medical Imaging, University of Toronto, Toronto, Canada

**Keywords:** Preoperative care, Thoracic radiographs, Pre-assessment, Trauma surgery, Proximal femur fracture, Hip fracture, Cost-effectiveness, Anesthesia, Additional value, Preparative screening, Geriatric

## Abstract

**Purpose:**

Preoperative screening in hip fracture patients is vital to minimize perioperative complications. Preoperative chest radiographs (POCR) are performed in many hip fracture patients. Earlier research showed that few POCR abnormalities influence perioperative policy. However, no studies in nonelective patient with a specific surgical conditions have been performed. With many hip fractures per year worldwide, a significant cost reduction could be made by performing selective POCR without compromising the quality of care. This study assessed the need for POCR in hip fracture patients.

**Method:**

Retrospective analysis of low-energy trauma patients was performed aged 18 years and older in the VU University Medical Center for a hip fracture in a 5-year period. All preoperative diagnostics were analyzed. All adjourned operations were evaluated.

**Results:**

A total of 642 patients were included, 70% female, matching current epidemiologic figures. The POCR showed abnormalities in 22.6%. In 0.6% the POCR lead to an adjournment of the operation (2.8% of abnormal POCR’s). These patients suffered from pneumonia. The POCR in these cases acted as a confirmation of the clinical diagnosis.

**Conclusion:**

Many factors involving the treatment of hip fracture patients are of importance in minimizing the risk of complications and mortality during and after admission. In 0.6% of all performed POCR’s an abnormality leads to the adjournment of the operation. In all four cases the POCR matched the clinical findings. Because the additional value of the POCR in hip fracture patients was limited, we think that its selective use in clinical abnormalities is safe and will reduce unnecessary costs.

## Introduction

Hip fracture patients are considered a frail group of patients and are seen on a daily basis in the Emergency Department (ED). Hip fractures occur at around 1.6 million per year worldwide, with this number expected to rise to 4.5 million by 2050 because of progressive aging [1]. It concerns an injury in need for operative treatment, due to the known and potential lethal complications. The 30-day mortality is high and is found to range between 7.5–13.3% [2, 3]. The cause of death is rarely due to the primary surgical condition, but as a result of medical complications during or after admission. The most common causes of 30-day mortality are pneumonia, sepsis and myocardial infarction [2].

Because of the frailty and the high postoperative mortality in this population the preoperative management is vital in order to identify correctable comorbidities and optimize the clinical condition of the patients as much as possible. The preoperative screening exists of a thorough history taking, physical examination, additional blood sampling, an electrocardiogram (ECG) and further additional testing if indicated. However, correcting comorbidities does cost time with possible worsening of patient’s condition due to immobilization. The timing of surgery remains vital but still some controversy exists. Unnecessary diagnostics with no consequences for the perioperative care that could delay surgery are potentially harmful and should be kept to a minimum.

Preoperative chest radiographs (POCR) are used in many hospitals in the preoperative setting, especially to rule out preexisting chest infections and evaluate existing disease to reduce the risk associated with anesthesia and surgery, increasing preoperative care quality [4]. However, the additional value of the POCR is under debate. An abnormal POCR can influence anesthetic and surgical management and can delay or adjourn the operation. The limited research on the POCR so far has shown that few POCR abnormalities demand a change in anesthetic or surgical management or correlate with a negative outcome during hospital stay in general surgical conditions [5]. Several studies [4–9] and more recent international guidelines [10, 11] suggest that the additional value of POCR is limited in patients undergoing elective non-cardiothoracic surgery and should only be performed when clinically indicated, in immigrants from developing countries without a chest radiograph in the last 12 months [4–10] or in patients scheduled for critical care [11]. Despite current recommendation the routine POCR is still frequently performed [4]. Because of the limited quality of evidence no clear recommendations can yet be made, and research is needed to identify the best strategy for specific surgical conditions in specific patient populations [4]. No studies have yet described the value of POCR with regard to the perioperative care in nonelective surgery patients or hip fracture patients specifically. Most of the hip fracture patients undergo POCR in the VU University medical center per protocol. With the rising age and rising incidence of hip fracture patients [1, 12], with a POCR costing €50, a significant cost reduction could be made by performing selective POCR without compromising the quality of perioperative care. This study assesses the additional value of routine POCR in hip fracture patients and will address the findings on the POCR, their influence on the perioperative management and the relation between per- and postoperative complications.

## Method

A retrospective analysis of a database of patients aged 18 years and older sustaining low-energy trauma (LET) treated by a trauma surgeon for a hip fracture in the VU University Medical Center (level 1 trauma center) within a five-year period (2008–2013) was performed. Patients sustaining a high-energy trauma (HET) and patients aged under 18 years were excluded.

Patient’s characteristics regarding age, gender, mechanism of injury, type of hip fracture, American Society of Anesthesiologist (ASA) score, cardiac and/or pulmonary medical history, type of operation and all per- and postoperative complications during hospital stay were registered. All preoperative diagnostics and consultancies were reported and analyzed. If an operation was adjourned, the indication was assessed. All data were obtained by retrospective patient chart reviewing. The POCR results were classified as either normal or abnormal by the first author based on the report made by the radiologist at the time. If judged abnormal, the abnormality was registered. Elongation and calcification of the aorta were judged as normal. Preoperative screening results were checked, and patients were declared fit for surgery by an anesthesiologist. The collected information was processed in a SPSS^®^20 database. Statistical differences were established by calculating the Pearson Chi-square.

## Results

A total of 642 patients met the inclusion criteria. 70.2% of the patients were female, and the median age was 83 years (IQR [77–89]) at the day of injury with incidence progressing with age, matching current epidemiologic figures [12]. ASA scores increased with age, as expected with rising comorbidities (see Table 1).

**Table 1:** Patient characteristics divided by normal and abnormal POCR

Characteristic	Normal POCR	Abnormal POCR	Total	*P* value
	*n* = 497	*n* = 145	*n* = 642	
**Age**				0.003
<50	20	0	20	
50–59	27	2	29	
60–69	52	9	61	
70–79	101	32	133	
80–89	207	61	268	
>90	90	41	131	
**Female**	104	347	451	0.659
**Type of trauma**				0.746
LET^a^	485	142	627	
Syncope	10	3	13	
Atraumatic	2	0	2	
**Type of hip fracture**				0.536
Intracapsular	260	70	330	
Intertrochanteric	215	70	285	
Subtrochanteric	22	5	27	
**ASA-classification**				0.000
I	52	5	57	
II	249	57	306	
II	163	72	235	
IV	11	9	20	
Missing	22	2	22	

*LET* Low-energetic trauma, *ASA* American Society of Anesthesiologist

^a^Cause of LET not always explored

The POCR showed abnormalities in 22.6% (145/642) of the cases (Table 2). Five patients were treated conservatively. Thirty-three of the 642 operations (5.1%) were adjourned (Table 3). In only 0.6% (4/642) of all cases the operation was adjourned as a result of abnormalities found on the POCR.

**Table 2:** Abnormalities shown on the POCR

	Freq.	%
Cardiomegaly	30	4.7
Emphysema	16	2.5
Widened mediastinum	12	1.9
Pulmonary edema	8	1.2
Pulmonary vein congestion	7	1.1
Splaying of the carina	7	1.1
Atelectasis	6	.9
Pleural effusion	5	.8
Fibrotic abnormalities	5	.8
Retrocardial consolidation	5	.8
Infiltrate^a^	4	.6
Rib fracture(s)	4	.6
(Possible) neoplasm	4	.6
Diafragmatic hernia	3	.4
Other	29	4.5
**Total**	**145**	**22.6**

^a^The only abnormality which lead to adjournment of the operation because of an abnormality on the POCR

**Table 3:** Reasons of adjournment of the operation

	Freq.	%
Abnormal laboratory value	18	2.8
Abnormal chest radiograph	4	.6
Logistic	2	.3
No operation because of infaust prognosis	2	.3
Ileus	2	.3
Abnormal ECG	2	.3
Diseased before operation	1	.2
Urine tract infections with fever	1	.2
Other	1	.2
**Total**	**33**	**5.1**

The abnormality found on the POCR in all four of the adjournments was concerned a pneumonia (indicated as infiltrate in Table 2). All four patients were aged 74 or higher, had ASA scores of three or higher and had elevated C-reactive protein (CRP) levels and white blood cell (WBC) counts in their blood samples besides the clinical symptoms of fever, coughing, shortness of breath and abnormalities during auscultation. With a POCR costing €50, approximately €32,000 could have been saved during this study period if a POCR was only made in these cases.

Out of the adjourned operations, one patient died before surgery because of a very poor pre-hospital condition with preexisting terminal heart failure.

Out of the eight POCR’s showing signs of pulmonary edema (Table 2), there were either clear clinical symptoms in all patients, new ECG abnormalities or no clinical influence of the pulmonary edema on the perioperative care. The four POCR’s that showed signs of neoplasm’s were all preexistent. Eleven of the abnormalities classified as other in Table 2 were consolidations, densities or nodules of unknown origin of which two abnormalities lead to CT scans during hospital stay, where one lung carcinoma was found.

### Consultancies

In five cases there were preoperative consultancies from the department of lung disease. These patients suffered from (possible) neoplasm’s, required evaluation of current known extensive lung and cardiac diseases or ruling out of a possible pulmonary embolism. One patient suffered from pneumonia in which the preoperative consultancy resulted in an adjournment of the operation.

### Time in hospital

The POCR did not lead to a delay in time till surgery with no statistical difference between the time from the ER to the OR in days and the results of the POCR (Table 4). Patients with an abnormal POCR did not have a statistical longer hospital stay than patients with a normal POCR.

**Table 4:** Summary of statistical calculations in relation with normal/abnormal POCR

Pearson Chi-square	Value	*df*	Asymp. Sig (2-sided)
Postoperative complications	0.760	1	0.383
Possible postoperative cardiothoracic complications	2.623	1	0.105
Time between ER and OR (days)	7.247	5	0.203

### Perioperative complications

There were no perioperative respiratory complications and only two minor perioperative cardiac complications (malpacing of a pacemaker and shortlasting ST-segment depression on a perioperative ECG).

### Postoperative complications

Postoperative complications occurred in 43.3% (274/633) of the patients during hospital stay. Of the 142 patients with an abnormal POCR, 46.6% (66/142) developed complications, compared to 42.4%(208/491) in the group with no abnormalities on the POCR. No statistical difference was found for either postoperative complications or any cardiothoracic-related complications in relation with the POCR findings (Table 4). The type of anesthesia did lead to a significant difference in postoperative complications. Patient with general anesthesia (GA) statistically had a higher chance of developing any postoperative complication and respiratory complications compared to spinal anesthesia (SA) (45.9 vs 36.9% with *P* = 0.037 and 7.7 vs 3.7% with *P* = 0.043, respectively). Twenty-six patients (4.1%) developed a pneumonia postoperatively (4.8% (20/220) in the GA compared to 3.2% (7/187) with no statistical difference. Thirteen patients died postoperatively of which four (31%) suffered from pneumonia. There were no statistical differences between GA and SA with regard to mortality. The result of the POCR did not influence the type of anesthesia. Complications or deaths did not differ between patients with intertrochanteric and femoral neck fractures, also when corrected for type of anesthesia. Five patients with an abnormal POCR died because of postoperative complications. Four of those are in no relation with the abnormality on the POCR and one patient with pleural effusion who died because of heart failure.

 Postoperative complications did differ between patients when classified by ASA score and the time till surgery (Table 5). ASA 1 patients were significantly more likely to develop any complications (12 vs 57%) when surgery was delayed over 24 h (*P* = 0.02), although small numbers featuring this group. There were no respiratory complications in the ASA 1 group. The disadvantage was not statistically seen in ASA 2 patients, although there were 10% more overall complications and 3% more respiratory complications in the group receiving surgery between 24–48 h versus surgery within 24 h. The patients that were classified as ASA 3 had a significant higher chance on developing postoperative complication and respiratory complications when surgery delay exceeded over 24 h. Although small numbers featured in the ASA 4 group, the risk of postoperative complications was not influenced by the timing of surgery. The timing of surgery did not influence mortality.

**Table 5:** Time between trauma and operation divided by ASA classifications and the risk of complications

ASA	Time between trauma and operation (h)	Any complication				Respiratory complications			
		Yes	No	Total	*P* value	Yes	No	Total	*P* value
1	<24	6	43	49		0	49	49	
	24–48	4	3	7		0	7	7	
	48–60	1	0	1		0	1	1	
	**Total**	11	46	57	0.02		57	57	–
2	<24	80	145	225		8	217	225	
	24–48	29	34	63		4	59	63	
	48–60	1	4	5		0	5	5	
	60–72	1	0	1		0	1	1	
	>72	5	5	10		1	9	10	
	**Total**	116	188	304	0.266	13	291	304	0.733
3	<24	74	78	152		9	143	152	
	24–48	45	15	60		13	47	60	
	48–60	4	4	8		2	6	8	
	60–72	1	4	5		0	5	5	
	>72	3	4	7		2	5	7	
	**Total**	127	105	232	0.005	26	206	232	0.004
4	<24	7	2	9		1	8	9	
	24–48	4	3	7		0	7	7	
	48–60	1	1	2		0	2	2	
	>72	2	0	2		0	2	2	
	**Total**	14	6	20	0.562	1	19	20	0.732
Total	<24	167	268	435		18	417	435	
	24–48	82	55	137		17	120	137	
	48–60	7	9	16		2	14	16	
	60–72	2	4	6		0	6	6	
	>72	10	9	19		3	16	19	
	**Total**	26	345	613	0.000	40	573	613	0.003

## Discussion

Many factors involving the treatment of hip fracture patients are of importance in minimizing the risk of complications and mortality during and after admission, but the additional value of POCR in hip fracture patients is limited, especially in patients with no clinical abnormalities prior to surgery. The POCR in our study showed abnormalities in 145 out of 642 (22.6%) patients with most of these being abnormalities matching with chronic diseases not requiring intervention. Only four out of these lead to an adjournment of the operation because of a chest infection. All four patients had clinical symptoms (fever, coughing, abnormal breath sounds during examination or elevated infection parameters) that supported the findings on the POCR. No significant differences in peri- and postoperative complications in relation with the result of the POCR were found.

Previous studies also suggest that the consequences of the abnormalities found on the POCR for scheduled operations are low, although only performed in elective patient groups. Guidelines of the European Society of Anesthesiology show that abnormal POCR only alter perioperative management in 3% out of the 23.1% of abnormal POCR’s [6]. Our study shows similar results and adds to the current evidence, being the first to describe the limited consequences of the POCR in a nonelective, fragile and specific surgical patient population.

The additional value of routine POCR is not only very limited but also associated with costs, potential delay of surgery because of unnecessary subsequent testing related to possible abnormal findings without any clinical relevance, causing worsening of the primary surgical condition and avoidable radiation risk for the patient [1]. An early intervention seems obvious since hip fractures lead to immobilization with many possible consequences. However, hip fracture patients often have significant comorbidities and a short delay to identify correctable ones in order to optimize their clinical condition is also logical. Mixed results are found when reviewing the ideal timing for surgery. An extensive review of the evidence for the ideal timing of surgery by Lewis et al suggests a trend for early operation within 12–48 h for ASA 1 and 2 patients [13]. Further delay over 48 h showed a rise in postoperative mortality. On the other hand, a delay up to five days for ASA 3 and 4 patients did not alter mortality. Though, a higher mortality rate was seen in patients of these categories when significant comorbidities were not corrected. However, the delay leaves the patient with significant pain and discomfort and the risk for pressure sores [13]. In our study a delay from 24 h onward in ASA 1, 2 (not significantly) and 3 patients resulted in more postoperative complications during admission, but did not alter mortality. There were no statistical differences showing an optimal window for ASA 4 patients. The outcome of POCR in general had no influence on the time till surgery.

The type of anesthesia is also an important factor. A recent study comparing RA versus GA in 328,540 matched surgical procedures showed that RA is associated with a 24% lower chance of respiratory complications, but did not alter 30-day mortality [14]. When comparing GA and RA in hip fracture patients, not only the incidence of postoperative respiratory complications decreases but also a 29% lower odds for mortality was seen in favor of the GA. Interestingly enough the advantages were only seen in patients with intertrochanteric fractures and not femoral neck fractures [15]. Our study also showed that patients undergoing GA were at higher risk for postoperative complications in general and especially respiratory complications. The differences based on fracture type were not found in our study.

Patients who are classified in a higher ASA group undergo GA and have a longer time till surgery independently have a higher chance of developing postoperative complications during admission. The POCR were not of any influence in these three factors.

There is always a chance of incidental findings on the POCR. One new incidental neoplasm (0.16%) was found during the study period.

The point should be raised that the retrospective design could have resulted in incomplete data collection. It was unknown how many patients were confused, which could influence the value of the preoperative screening because of difficult history taking. The follow-up of patients was limited to the time in hospital. The single center design does possibly limit the generalizability of this study. However, it can be reasoned that the geriatric population with a hip fracture does not differ between hospitals and therefore multicenter studies might not add more new evidence.

Some patients will need postoperative chest radiographs, and a possible benefit of the POCR is that it provides a comparative film. Some POCR’s will also act as a reassurance when there is doubt over the clinical condition of a patient. However, if the condition of the patient is doubtful or changes postoperatively, a chest radiograph is indicated without the need for a comparative film.

The interobserver variation in interpretation of the POCR has not been taken into account in this study. False negative results and false positive results can lead to per- and postoperative complications and unnecessary and potential harmful diagnostics and extended hospital stay, respectively. However, in this way the human error during daily practice is simulated.

The incidence of abnormal POCR rises with age [2]. The age of patients whose operation was adjourned because of an abnormal POCR was all above 70 years with ASA scores three or higher. Based on these data we cannot assess whether there are other groups of patients, besides symptomatic patients, who have an indication for a POCR since the greater part of all patients in this study are aged above 70 years with high ASA scores.

Most patients with abnormalities on the POCR that could have influenced perioperative care had either clinical symptoms or abnormal preoperative diagnostic tests. Even though this study had a retrospective design, it can be reasoned that performing selective POCR in case of clinical abnormalities can be a safe practice for patients with a (LET) hip fracture.

## Conclusion

The value of the POCR in patients with a hip fracture is limited. Only in 0.6% of all performed POCR’s an abnormality leads to the adjournment of the operation. In these cases, the POCR matched the clinical findings. The known risk factors (GA, longer time till surgery and higher ASA classifications) for developing postoperative complications were not influenced by the POCR. The result of the POCR did not influence the rate of postoperative complications or mortality, and there were no cardiothoracic-related perioperative complications related to the results of the POCR. The preoperative screening in hip fracture patients is vital, though we think that the selective use of POCR in clinical abnormalities is safe and will reduce unnecessary delay and costs without compromising the quality of perioperative care.
